# Contrasting Geographical Distributions as a Result of Thermal Tolerance and Long-Distance Dispersal in Two Allegedly Widespread Tropical Brown Algae

**DOI:** 10.1371/journal.pone.0030813

**Published:** 2012-01-26

**Authors:** Ana Tronholm, Frederik Leliaert, Marta Sansón, Julio Afonso-Carrillo, Lennert Tyberghein, Heroen Verbruggen, Olivier De Clerck

**Affiliations:** 1 Phycology Research Group and Centre for Molecular Phylogenetics and Evolution, Ghent University, Ghent, Belgium; 2 Departamento de Biología Vegetal (Botánica), Universidad de La Laguna, La Laguna, Canary Islands, Spain; Brigham Young University, United States of America

## Abstract

**Background:**

Many tropical marine macroalgae are reported from all three ocean basins, though these very wide distributions may simply be an artifact resulting from inadequate taxonomy that fails to take into account cryptic diversity. Alternatively, pantropical distributions challenge the belief of limited intrinsic dispersal capacity of marine seaweeds and the effectiveness of the north-south oriented continents as dispersal barriers. We aimed to re-assess the distribution of two allegedly circumtropical brown algae, *Dictyota ciliolata* and *D. crenulata*, and interpret the realized geographical range of the respective species in relation to their thermal tolerance and major tectonic and climatic events during the Cenozoic.

**Methodology/Principal Findings:**

Species delimitation was based on 184 chloroplast encoded *psb*A sequences, using a Generalized Mixed Yule Coalescent method. Phylogenetic relationships were inferred by analyzing a six-gene dataset. Divergence times were estimated using relaxed molecular clock methods and published calibration data. Distribution ranges of the species were inferred from DNA-confirmed records, complemented with credible literature data and herbarium vouchers. Temperature tolerances of the species were determined by correlating distribution records with local SST values. We found considerable conflict between traditional and DNA-based species definitions. *Dictyota crenulata* consists of several pseudocryptic species, which have restricted distributions in the Atlantic Ocean and Pacific Central America. In contrast, the pantropical distribution of *D. ciliolata* is confirmed and linked to its significantly wider temperature tolerance.

**Conclusions/Significance:**

Tectonically driven rearrangements of physical barriers left an unequivocal imprint on the current diversity patterns of marine macroalgae, as witnessed by the *D. crenulata*–complex. The nearly circumglobal tropical distribution of *D. ciliolata*, however, demonstrates that the north-south oriented continents do not present absolute dispersal barriers for species characterized by wide temperature tolerances.

## Introduction

Geographical distributions and range sizes of species typically result from the complex interplay of ecological and historical factors. The combined effect of an organism's ability to disperse and the strength of dispersal barriers will, amongst other factors, greatly influence the realized distribution of a species. The apparent lack of dispersal barriers combined with high dispersal capacities has often been invoked to explain wide distribution ranges of many marine species [1], [2]. However, even among marine organisms distribution ranges vary widely as do their intrinsic dispersal capacities. Kinlan & Gaines [3], using isolation-by-distance slopes, determined that propagule dispersal varies at least over five orders of magnitude among marine organisms. Compared to marine invertebrates and fish, macroalgae are considered poor dispersers. Even though manifest evidence for long distance dispersal exists, these events are probably relatively uncommon [4]–[7]. Congruent with limited dispersal capacities, accumulating molecular data provide evidence for the prevalence of geographically restricted cryptic species in many allegedly globally distributed seaweeds e.g. [8]–[10].

For tropical marine coastal organisms the closure of the circumtropical Tethyan seaway and the North-South orientation of the African and American continents together with a steepening of the sea surface temperature gradient from the equator to the poles since the late Eocene [11] resulted in major dispersal barriers. Sea surface temperature is known to play a pivotal role in determining the range and position of distributions of macroalgae, and marine organisms in general e.g. [12]–[15]. Temperature limits for survival, growth and/or reproduction correlate well with the geographic ranges of seaweeds [16]. Most species display a rather narrow thermal tolerance range which is reflected in restricted latitudinal distributions [17]–[19]. For tropical species this implies a serious constraint on dispersal between oceanic basins. Alternatively, and avoiding cold water dispersal barriers, interoceanic dispersal may succeed through the Suez and Panama Canal, provided that the species cope with the osmotic stress resulting from the hyper- or hyposaline conditions that characterize these canals. Despite the apparent strength of the barriers that separate the major ocean basins, unequivocal molecular evidence for widespread circumtropical algal distributions has been presented for some seaweeds, including *Caulerpa* species [20]–[21], the *Boodlea*-complex [22] and the red alga *Murrayella periclados* [23]. These distributions challenge the belief of limited intrinsic dispersal capacity and the effectiveness of the north-south orientated continents as dispersal barriers for tropical marine seaweeds.

In this study we address the genetic differentiation and distribution patterns of *Dictyota*, a prominent genus in tropical to warm-temperate oceans. We focus on *Dictyota* species with dentate margins, which are generally reported under the names *D. ciliolata* and *D. crenulata*. *Dictyota ciliolata*, originally described from the Caribbean Sea, is regarded as an illustrative species with a pantropical distribution [24]–[29]. *Dictyota crenulata*, originally described from southern Pacific Mexico, closely resembles *D. ciliolata* but differs in the abundance and shape of the marginal teeth. In *D. crenulata* the margins are set with numerous triangular-shaped teeth that are regularly spaced; while in *D. ciliolata* teeth are much less abundant and irregularly spaced or absent altogether. Like *D. ciliolata*, *D. crenulata* has been assumed to be broadly distributed in the tropics, although its occurrence in the Indo-West Pacific has been questioned [27]. In the Caribbean Sea the species is frequently reported as *D. jamaicensis* Taylor, a taxon which is considered synonymous with *D. crenulata* [30].

Since these very wide distributions may simply be an artifact resulting from inadequate taxonomy which fails to take into account cryptic diversity we aim to: 1. delimit species using a sequence-based algorithmic methodology based on a dataset of 184 chloroplast encoded *psb*A sequences of *Dictyota* representatives; 2. reinterpret geographical distributions of the respective species; 3. assess how temperature tolerance and the closure of the Tethyan seaway in the Cenozoic have shaped the current ranges and diversity patterns using a phylogenetic approach. Temperature tolerances of the species were determined by correlating distribution records with sea surface temperature data.

## Materials and Methods

### Taxon sampling and DNA sequencing

We sampled an extensive number of specimens of *Dictyota ciliolata*, *D. crenulata* and other *Dictyota* species collected worldwide (see Table S1 in Supporting Information). Morphological species identification was based on regional floras and a recent taxonomic treatise of the genus [24], [27], [30]–[32]. Total genomic DNA was extracted from silica gel preserved material using a standard CTAB-extraction method and subsequent purification with a Wizard® DNA Clean-Up System (Promega Inc., Madison, WI, USA) as outlined in De Clerck et al. [33]. The plastid-encoded *psb*A (photosystem II reaction center protein D1) and *rbc*L (RuBisCO large subunit) genes were amplified and sequenced as outlined by De Clerck et al. [33] and Hwang et al. [34]. Mitochondrial *cox*1, *cox*3 and *nad*1 genes were amplified and sequenced according to Tronholm et al. [35]. The protein coding sequences were aligned by eye using MEGA 5 [36]. A complete list of specimens used in the molecular analyses is detailed in Table S1 in Supporting Information.

### Species delimitation

Species were delimited using a *psb*A dataset of 184 unique *Dictyota* sequences. We used an algorithmic approach developed by Pons et al. [37] and Monaghan et al. [38]. The method, using a Generalized Mixed Yule Coalescent (GMYC) model aims to detect the transition between micro- and macroevolutionary patterns using an ultrametric tree and hence define the species boundary. A maximum likelihood approach is used to optimize the shift in branching rates in an ultrametric gene tree from interspecific branches (Yule model) to intraspecific branches (neutral coalescent). To obtain an ultrametric tree, a Bayesian phylogenetic analysis, using one sequence for each haplotype, was conducted in BEAST v1.5.3 [39] under a GTR+ I+G model with an uncorrelated lognormal (UCLN) relaxed molecular clock model [40] and using a coalescence tree prior. Two Markov Chain Monte Carlo (MCMC) analyses were run for 10 million generations, sampling every 1000^th^ generation. The output was diagnosed for convergence using Tracer v.1.5, and summary statistics and trees were generated using the last five million generations with TreeAnnotator v1.5.3 [41]. GMYC analyses were performed under single- and multiple-threshold models [38], using the SPLITS package for R (http://r-forge.r-project.org/projects/splits/). Inter- and intraspecific uncorrected p-distances were calculated in MEGA 5 [36].

### Phylogenetic analyses

A multigene phylogenetic analysis was based on a data matrix of 35 *Dictyota* species and 6 genes (*rbc*L, *psb*A, *nad*1, *cox*1, *cox*3, LSU rDNA; see Table S2 in Supporting Information). The 35 *Dictyota* species correspond to separately evolving lineages derived from the GMYC analyses performed on the *psb*A data set. The dictyotalean genera *Canistrocarpus*, *Dictyopteris*, *Dilophus*, *Padina*, *Rugulopteryx*, *Scoresbyella* and *Spatoglossum* were used as outgroup. Model selection and partitioning strategy follow Tronholm et al. [35]. The Bayesian information criterion (BIC) was used as the selection criterion. The guide tree used during the entire procedure was obtained by maximum- likelihood (ML) analysis of the unpartitioned concatenated alignment using a JC+Г_8_ model. All subsequent likelihood optimizations and BIC calculations were carried out with Treefinder [42]. The partitioning strategy plus model combination that received the lowest BIC score was used in the phylogenetic analyses. Maximum likelihood (ML) searches were carried out with Treefinder [42] using seven partitions [LSU rDNA (one partition); plastid and mitochondrial genes partitioned according to codon position (2×3 partitions)], and a GTR model with gamma distribution and four rate categories (GTR+I+G) per partition. Branch support was calculated by non-parametric bootstrapping (1000 replicates). Bayesian phylogenetic inference (BI) was carried out with MrBayes 3.1.2 [43] using the same partitions and models, and default priors. Two parallel runs, each consisting of four incrementally heated chains were run for 15 million generations, sampling every 1000^th^ generation. Convergence of log-likelihoods and parameter values was assessed in Tracer v1.5 [41]. A burnin sample of 1000 trees was removed before constructing the majority rule consensus tree.

### Time-calibrated phylogeny

A cautious attempt is made to establish a time-frame of diversification in *Dictyota* by inferring a chronogram based on the same multigene alignment in BEAST. In the absence of reliable *Dictyota* fossils, two nodes in the tree were constrained in geological time based on a previously published brown algal time-calibrated phylogeny [44]. The split between *Padina* and the *Dictyota*-*Dictyopteris* clade was constrained at 99.6–129 Ma, and the split between the latter two genera was constrained at 68.8–113.5 Ma, both with uniform priors. The analysis was performed under a GTR+ I+G model with an uncorrelated lognormal (UCLN) relaxed molecular clock model, using a uniform tree prior. Four independent runs of 20 million generations each were run sampling every 10000^th^ generation. Convergence and stationarity of the chains were evaluated in Tracer v1.5. The majority rule consensus tree was based on 7200 trees sampled across a large part of the four runs.

### Thermal tolerance

Thermal preferences and tolerance limits were estimated by plotting distribution records on geographic information system (GIS) maps of environmental variables. We used Bio-ORACLE [45], a dataset providing marine environmental information for global-scale applications, to extract sea surface temperature (SST) data (minimum, maximum and mean). Occurrence records are primarily based on recently collected specimens for which the identification has been confirmed by DNA sequence information. These records are complemented with verified literature data and herbarium specimens that were carefully re-examined by the first and last author. Recent collections had accurate coordinates recorded with a global positioning device. Older collections with detailed locality information were georeferenced (latitude and longitude) using Google Earth (http://earth.google.com).

To define the species' thermal biology we pooled the realistic geographical distribution of the species with their respective temperature tolerances. The ‘maximum thermal tolerance range’ was calculated as the largest difference between the maximum and the minimum sea surface temperature based on the species' occurrence records, as these represent the most ecologically realistic measure of a species' tolerance of high and low temperatures.

## Results

### Species delimitation

**Figure 1:**
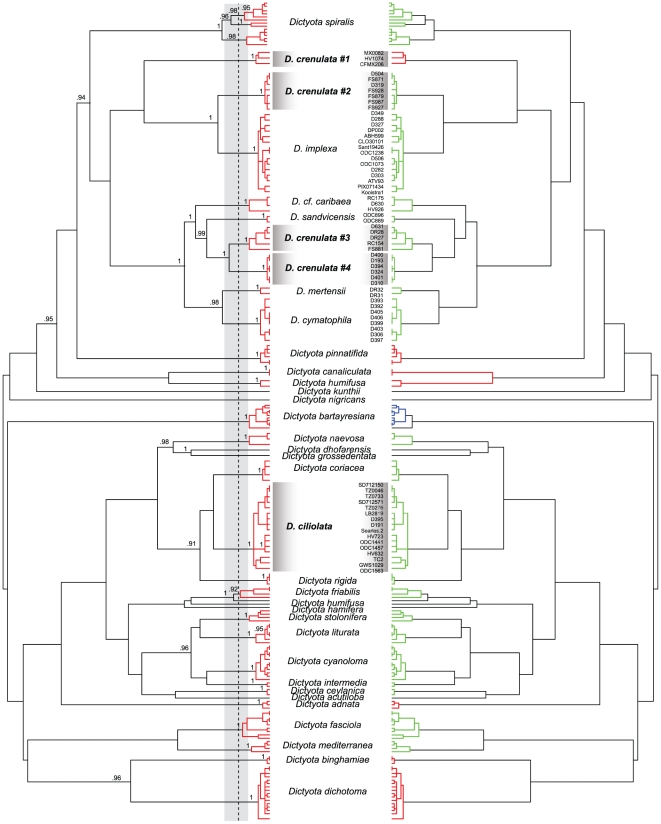
Ultrametric tree of *Dictyota* based on a Bayesian analysis of 184 *psb*A sequence data. Divergence times were estimated under a relaxed molecular clock using an uncorrelated lognormal (UCLN) model in BEAST. The dotted vertical line indicates the maximum likelihood transition point of the switch in branching rates, as estimated by a general mixed Yule-coalescent (GMYC) model. The GMYC analysis was performed using a single threshold (left) and multiple thresholds (right).

We analysed branch length dynamics in the ultrametric *psb*A tree to delimit species. The likelihood of the GMYC model was significantly higher than that of the null model of uniform (coalescent) branching rates (Table 1). Using the single-threshold GMYC, the depth (T) from the branch tips at which the transition occurred was 0.00469 substitutions per site. The model estimates 39 putative species, with a confidence interval ranging from 34 to 43. The multiple-threshold model detected the same number of putative species, although with a markedly broader confidence interval, from 21 to 39. *Dictyota ciliolata* and *D. crenulata* were resolved in five GMYC lineages under both models (Fig. 1). *Dictyota crenulata* consists of four GMYC lineages: one Pacific Central American lineage, two Macaronesian lineages and one amphi-Atlantic lineage. Several non-dentate *Dictyota* species, *Dictyota* cf. *caribaea*, *D. cymatophila*, *D. implexa*, *D. mertensii* and *D. sandvicensis*, also fall within this *D. crenulata* clade. In contrast to *D. crenulata*, all specimens identified as *D. ciliolata* form a single GMYC lineage, which also includes the morphologically allied *D. menstrualis* and *D. plectens*. Sequence divergence, calculated as uncorrected *p*-distances (see Figure S1 in Supporting Information), within these putative species ranged from complete identity to 0.8% with 95% of the values equal or lower than 0.7%. Distances among species ranged from 1% to 10.2%.

**Table 1:** Lineage branching pattern fit to single- and multiple threshold variants of the GMYC model.

Model	T	N_GMYC_	CI	L_0_	L_GMYC_	LR
single	0.00469	39	34–43	1649.088	1671.926	45.67581*
multiple	-	39	21–39	1649.088	1674.306	50.43415*

T, threshold genetic distance from the branch tips where transition occurred (presented for single-threshold models).

N_GMYC_, number of putative species as the sum of sequence clusters and singletons, CI, confidence intervals as solutions within 2 log-likelihood units of the maximum likelihood.

L_0_, likelihood for null model (the same for single and multiple threshold model comparisons.

L_GMYC_, likelihood for GMYC model.

LR, significance of the likelihood ratio evaluated using a chi-square test with 3 degrees of freedom to compare GMYC and null models.

*p < 0.001.

### Phylogenetic analyses

The concatenated alignment of six genes consisted of 54 species and 5487 nt (LSU rDNA = 1290 bp; *psb*A = 885 bp; *rbc*L = 1293; *cox*1 = 645 bp; *cox*3 = 657 bp; *nad*1 = 717 bp). The matrix was 77% filled (see Table S2 in Supporting Information). ML and BI yielded virtually identical tree topologies and the nodes within the clades of interest (*D. ciliolata* and *D. crenulata* clade) were well supported. The phylogenetic tree obtained from the ML analysis (ln*L* = −45960.04), with indication of ML bootstrap values and BI posterior probabilities, is shown in Figure S2 in Supporting Information. The *D. ciliolata* and *D. crenulata* clades do not form a monophyletic assemblage. The *D. ciliolata* clade is sister to *D. coriacea* and *D. acutiloba* with moderate support, while the *D. crenulata* clade is sister to *D. pinnatifida* and *D. spiralis*.

**Figure 2:**
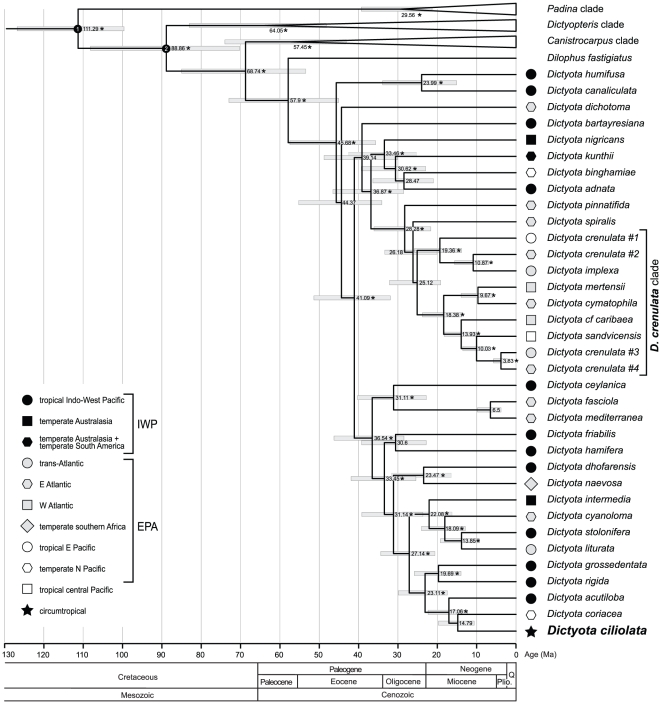
Time-calibrated phylogeny. Highest density probability (95% HPD) intervals are provided for each node as well as the posterior probabilities, stars represent strong support (p.p.>0.95).

An Eocene origin of the genus *Dictyota* was inferred, 46 (95% HPD: 35–57) Ma (Fig. 2). The backbone of the genus *Dictyota* is characterized by a radiation of lineages which received no support. The *D. crenulata* complex may have gradually diverged from the late Oligocene onwards (23, 95% HPD: 18–32 Ma), with the most recent divergence recovered between *D. crenulata*#3 and *D. crenulata*#4 (2.6, 95% HPD: 2.2–5.8 Ma). *D. ciliolata* may have diverged from *D. coriacea* ca. 14 Ma.

### Geographical distribution

**Figure 3:**
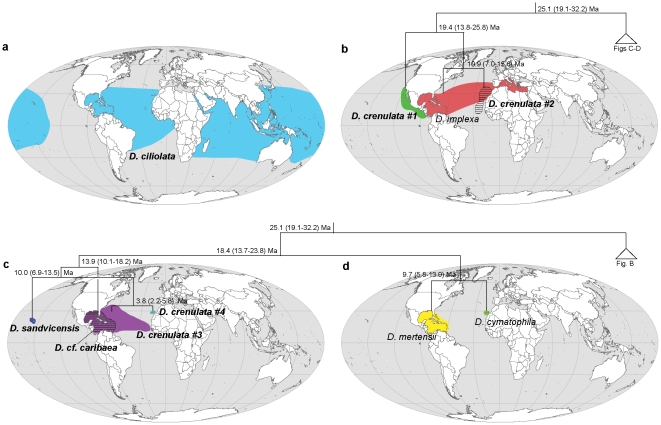
Distribution maps. Distribution of the species belonging to *D. ciliolata* (a) and the *D. crenulata*-complex (b–d) with superimposition of phylogenetic relationships.

We determined the geographical distribution of the species on the basis of DNA-confirmed records, complemented with credible literature data and re-examined herbarium specimens. *Dictyota ciliolata* is broadly distributed in the tropical to subtropical Atlantic and Indo-West Pacific. The northernmost DNA-confirmed record is located in North Carolina and the southernmost in Carnac Island in Western Australia. Genuine *D. crenulata* (*D. crenulata*#1) is restricted to the Pacific coast of Central America, from Costa Rica to Baja California. Non-Pacific specimens morphologically identified as *D. crenulata*, resolved as three separate species, which are all confined to the Atlantic Ocean. *Dictyota crenulata*#2 occurs in the Canary Islands, Madeira and Cape Verde. *Dictyota crenulata*#3 has an amphi-Atlantic distribution occurring in the Caribbean Sea, Bermuda and Cape Verde Islands. *D. crenulata*#4 is only known from the Canary Islands. The non-dentate members of the *D. crenulata* clade, *D. implexa*, *Dictyota* cf. *caribaea*, *D. mertensii*, *D. cymatophila* and *D. sandvicensis* show similar geographically confined distributions, with only *D. implexa* having an amphi-Atlantic distribution (Fig. 3).

### Species distribution ranges and thermal tolerance

**Figure 4:**
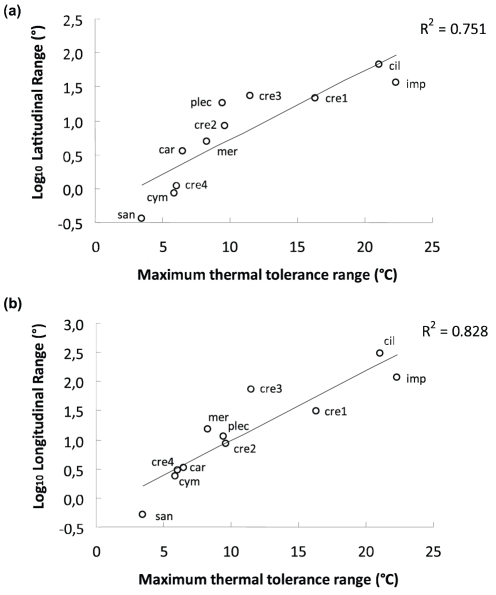
Correlation between maximum thermal tolerance range (°C) and log-transformed latitudinal range (A), and log-transformed longitudinal range (B). The correlation coefficient (R^2^) is shown for each case. Symbols represent individual species' as follows: *Dictyota* cf. *caribaea* (car), *Dictyota ciliolata* (cil), *Dictyota crenulata*#1 (cre1), *Dictyota crenulata*#2 (cre2), *Dictyota crenulata*#3 (cre3), *Dictyota crenulata*#4 (cre4), *Dictyota cymatophila* (cym), *Dictyota implexa* (imp), *Dictyota mertensii* (mer), *Dictyota sandvicensis* (san).

*Dictyota* species varied widely in their latitudinal as well as longitudinal range (Table 2). Mean values of thermal tolerance ranged from 21.1°C in *D. implexa* to 28.5°C in *Dictyota* cf. *caribaea*. Differences in maximum and minimum values of SST between species were more pronounced, which were also shown by these two species (31°C in *Dictyota* cf. *caribaea* and 17.1°C in *D. implexa*). Significant correlations were detected between maximum thermal tolerance range (°C) and latitudinal range (R^2^ = 0.751), and longitudinal range (R^2^ = 0.828), i.e. species with the highest thermal tolerance showed broader latitudinal and longitudinal ranges (e.g. *D. implexa* and *D. ciliolata*) (Fig. 4).

**Table 2:** Summary of geographic range and Sea Surface Temperature (SST) for each species.

Species	n	Lat range (°)	Lon range (°)	Max (°C)	Mean (°C)	Min (°C)	Max range (°C)
*Dictyota* cf. *caribaea*	3	3.6	3.3	31.0	28.5	26.1	6.6
*Dictyota ciliolata*	305	66.8	308.5	29.9	27.5	24.9	21.1
*Dictyota crenulata*#1	25	21.1	30.9	29.4	25.9	22.6	16.3
*Dictyota crenulata*#2	18	18.1	11.2	26.2	23.6	21.5	9.5
*Dictyota crenulata*#3	64	23.0	73.2	29.6	27.7	25.9	11.5
*Dictyota crenulata*#4	14	1.1	2.9	23.8	21.2	18.9	6.1
*Dictyota cymatophila*	13	0.9	2.3	23.8	21.3	18.9	5.9
*Dictyota implexa*	111	36.3	116.4	25.9	21.1	17.1	22.3
*Dictyota mertensii*	15	4.9	15.3	30.2	27.9	26.3	8.3
*Dictyota sandvicensis*	13	0.4	0.5	27.1	25.7	24.4	3.4

n = number of records.

Lat range = latitudinal range.

Lon range = longitudinal range.

Max = mean of the maximum SST.

Mean = mean SST.

Min = mean of the minimum SST.

Max range = range between the maximum and the minimum SST values.

## Discussion

### Species boundaries and geographic range

Molecular screening of geographically disparate populations of a common tropical *Dictyota* species, *D. crenulata*, revealed a complex of multiple pseudocryptic species, which also include several other species. In contrast, results indicated that the widespread tropical species *D. ciliolata* consists of a single species. The contrasting results for *D. crenulata* and *D. ciliolata* illustrate the difficulties related to using morphology as an estimator of species diversity in structurally simple organisms. This is especially evident when these organisms are also characterized by a considerable degree of morphological plasticity, as is the case in *Dictyota*. Species delineation can therefore be best achieved by the analyses of gene sequence data. In this study we apply an algorithmic species delineation approach to redefine species boundaries. Both the original GMYC model developed by Pons et al. [37] and the modified version that allows for a variable transition from coalescent to speciation among lineages [38] converge on the same number of independently evolving lineages and the specimens attributed to the respective lineages were fully congruent in both analyses. Values of uncorrected interspecific distances fall completely within the range of distances reported in the literature for the *psb*A gene in Dictyotales [46]–[48] and lend further support to the application of GMYC modeling to delineate independently evolving lineages. *Dictyota crenulata* illustrates well how misconceptions about species boundaries impact on our understanding of distributions and historical biogeography of tropical seaweeds. Once properly delineated, the taxa making up the *D. crenulata*-clade have much more restricted geographic ranges, being exclusively confined to either the Atlantic or the Eastern Pacific Ocean.

Contrary to *Dictyota crenulata*, where geographically disparate populations segregate as distinct evolutionary lineages, results showed that the widespread tropical species *D. ciliolata* constitutes a single evolutionary lineage. This lineage also includes specimens traditionally attributed to *D. menstrualis* and *D. plectens* from the warm temperate western Atlantic (North Carolina) and south-east Pacific (Lord Howe Island), respectively. The *D. ciliolata* clade comprises four subclades segregating according to geographical origin: an Indo-Pacific subclade containing specimens from Indonesia as well as East Africa, a subclade with specimens from the Philippines, an Atlantic subclade with species from both sides of the Atlantic, and a clade uniting the specimens identified as *D. plectens* together with one specimen from East Africa. The fact that samples of *D. ciliolata*, *D. menstrualis* and *D. plectens*, regardless of geographic origin, do not represent separately evolving units is highly relevant given the observation of Lohse [49] who demonstrated that the General mixed-Yule coalescent model has a tendency to overestimate species numbers, especially when sampling of intraspecific variation is low or uneven. With our moderate taxon sampling (20 specimens sequenced and 12 unique haplotypes) and given the vast geographic range of *D. ciliolata* it is highly unlikely that the entire genetic variation of the species was adequately sampled. Nevertheless, GMYC model does not consider these subclades as separately evolving. This leads us to believe that *D. ciliolata* (incl. *D. menstrualis* and *D. plectens*) maintains genetic cohesion over geographic scales spanning several ocean basins.

### Diversification and historical biogeography

The paucity of fossil records has impeded the estimation of divergence time estimates in brown algae. Silberfeld et al. [44] presented the first time-calibrated phylogeny of the Phaeophyceae, which was based on a multigene dataset constrained with three fossil calibration points. The chronogram presented in this study is based on these divergence estimates for the Dictyotales and may be treated as a first attempt to assess the evolutionary history of the genus *Dictyota*. Therefore, and given the limited number of fossil constraints, the divergence estimates presented here need to be interpreted with care. The origin of the genus *Dictyota* is inferred to be ca. 40 Ma (95% HPD: 31–52 Ma). Initial diversification appeared to be very rapid, with several lineages emerging nearly simultaneously.

The *D. crenulata* clade seems to have diverged gradually from the late Oligocene onwards (>25 Ma) with the most recent inferred divergence dated at ca. 3 Ma. The early splits in the Eastern-Pacific-Atlantic clade (EPA-clade) show no obvious signature of a vicariant event across the Central American Isthmus. The putative earliest diverging lineages, *D. spiralis* and *D. pinnatifida* have an exclusively Atlantic distribution and Pacific – Atlantic sister relationships are to be found only between *D. crenulata*#1 – *D. implexa*/*crenulata*#2 and *Dictyota* cf. *caribaea* – *D. sandvicensis*. The diversification estimates of these sister lineages predate the effective closure of the Tropical American Seaway (3.1 Ma). Similar observations have been reported for many marine organisms including seaweeds [50]–[53]. Although we cannot rule out that our molecular clock analysis overestimates divergence times, it is likely that the final closure of the Panama Isthmus was not the initial cause of divergence between populations. The uplift of the Panama Isthmus was a complex process that took place over at least 12 Ma before completion at ca. 3 Ma [54], [55], and the final closure of the land bridge may have acted as barrier reinforcement after divergence had been initiated by oceanographic events [50]–[52]. Moreover, the eventual uplift of the Panama Isthmus was followed by major extinctions in the western Atlantic [56], which can lead to overestimation of divergence times since trans-isthmian species pairs may not be true sisters [50], [52].

### Speciation and dispersal in the Atlantic Ocean

Diversification in the Atlantic Ocean most likely involved repeated peripatric speciation events that took place in the Miocene and Pliocene epochs. Peripatric speciation involves founder events resulting from long distance dispersal of a small number of individuals and subsequent genetic differentiation of the established population [57]. For this mode of speciation to qualify as founder speciation, depends on the frequency and temporal variation of dispersal events. Paulay & Meyer [2] argue that if dispersal is very rare at all times founder speciation is favored, while long-term temporal variation of dispersal favors vicariance. Because of the vast distance that separates the Western from the Eastern Atlantic and the lack of intermediate suitable substrate, one would intuitively favor founder speciation when considering species pairs on both sides of the Atlantic. The presence of two amphi-Atlantic species, *D. implexa* and *D. crenulata*#3, however, illustrates ongoing dispersal and connectivity across the Atlantic Ocean. Although DNA-confirmed data for marine benthic algae are scarce, similar distribution patterns of amphi-Atlantic species of the green algal genera *Cladophoropsis* [58], [22] and *Halimeda* [59], [60] may be indicative for relatively high gene flow across the Atlantic Ocean and give more credibility to vicariant speciation. More detailed studies applying more variable markers and including greater taxon sampling could shed important insights on the phylogeographic structure of these algae, quantify gene flow and directionality across the ocean basin.

### Dispersal and thermal tolerance

The broad geographical range sizes of *D. ciliolata* and some species in the *D. crenulata* clade suggest high dispersal potential. This is in contrast with many other attached seaweed species that have more restricted geographical ranges, either as a consequence of narrow ecological tolerance resulting in habitat unsuitability (phenotype-environment mismatches) or effective dispersal limitation [19], [7], [53]. An effective way for *Dictyota* to disperse might be by small thallus fragments or microthalli that are able to drift in the water column, re-attach and grow successfully at new sites [61]. In addition, fertile thallus fragments may drop propagules on arrival [4], [6], [22].

The asymmetrical biogeographies of *Dictyota ciliolata* and the segregated *D. crenulata* species are congruent with the widely accepted view that thermal tolerance is an important factor in determining latitudinal range size of marine as well as terrestrial organisms [13]–[15], [62], [63]. While *D. ciliolata* has been successful in expanding its range throughout the tropical to warm-temperate Atlantic and Indo-West Pacific, the segregated *D. crenulata* species have more restricted distributions that are separated to partly overlapping within a single ocean basin (Atlantic or Eastern Pacific Oceans). Our data suggest that the biogeographical asymmetry between *D. ciliolata* and the species in the *D. crenulata* clade can best be explained by differences in thermal tolerance range, enabling or restricting dispersal over cold-water barriers.

Sea surface temperature along the south-west African coast steeply declined by the appearance of the Benguela upwelling system in the late Miocene [64]. Along with this cold-water barrier, the formation of the Levant in the early Miocene [65] and the Messinian Salinity Crisis in the late Miocene resulted in a strong dispersal barrier for tropical marine organisms between the Atlantic and Indo-West Pacific. The nearly circumtropical distribution of *D. ciliolata* likely results from its tolerance towards relatively low minimum temperatures, allowing it to cross southern Africa's cold-water barrier, followed by successful dispersal and establishment throughout the Atlantic and Indo-West Pacific Oceans.

Although the cold water Benguela upwelling system first appeared in the Miocene, it did not become a permanent feature until the late Pliocene [66], [67], but even in recent times, eddies of the warm Agulhas current (‘Agulhas rings’) pass into the tropical South Atlantic, possibly allowing for occasional migration of marine tropical organisms from the Indian Ocean to the Atlantic [13], [68]. Another possible corridor for recent dispersal between the Atlantic and the Indo-West Pacific is the Suez Canal, which opened in the mid 1800s. Several studies have provided evidence for colonization of species from the Red Sea to the Mediterranean Sea (Lessepsian migration), with scarcely any evidence of dispersal in the opposite direction [69]. *Dictyota ciliolata* occurs in the Red Sea, but its absence from the Mediterranean Sea [35] makes Lessepsian migration less plausible. Despite high dispersal potential, *D. ciliolata* is absent from the eastern Pacific Ocean. Trans-Pacific dispersal of coastal organisms is limited by the vast expanse of the eastern Pacific Ocean, and only a few species occur on both sides of this eastern Pacific Barrier [70], [71]. The East Pacific also likely acts as a strong barrier for *Dictyota* species with tropical affinities. The isolated Hawaiian occurrence of *D. sandvicensis*, which diverged from the Atlantic species pair *D. crenulata*#3 and *D. crenulata*#4 ca. 8 Ma, is more difficult to explain but may have resulted from a peripatric speciation event. As has been suggested for several groups of marine organisms, the eastern Pacific Barrier acts as a haphazard filter allowing sporadic dispersal events that are separated by periods of time long enough to cause speciation.

## Supporting Information

Figure S1 Estimates of evolutionary divergence (uncorrected p-distances) between *psb*A sequences, using unique haplotypes only. Raw data available upon request.
(EPS)

Figure S2 Multigene phylogeny. Phylogenetic hypothesis (lnL = −45960.04) obtained by maximum likelihood inference of a dataset containing six genes (partial LSU rDNA, *rbc*L, *psb*A, *cox*1, *cox*3 and *nad*1). Numbers at the nodes indicate ML bootstrap values followed by posterior probabilities; values below respectively 50 and 0.7 are not shown.
(EPS)

Table S1 Specimens used in the molecular analyses with indication of collecting data. In the first column, (•) indicates specimens used for the species delimitation analyses and (○) indicates the specimens used for the multigene phylogenetic analyses.
(PDF)

Table S2 Genbank accession numbers of the sequences used in the concatenated alignment, including strain numbers and sequence length.
(PDF)
