# Supplementary material for: Contrasting Geographical Distributions as a Result of Thermal Tolerance and Long-Distance Dispersal in Two Allegedly Widespread Tropical Brown Algae
Source: PLoS One. 2012 Jan 26;7(1):e30813. doi: 10.1371/journal.pone.0030813 (PMC3266907; doi:10.1371/journal.pone.0030813)
Supplement: Table S1 — Specimens used in the molecular analyses with indication of collecting data. In the first column, (•) indicates specimens used for the species delimitation analyses and (○) indicates the specimens used for the multigene phylogenetic analyses. (PDF) [file pone.0030813.s003.pdf]

Table S1. Specimens used in the molecular analysis with indication of collecting data and Genbank accession numbers of the sequences used (see Table S2 for multiple accession numbers of sequences related to a single specimen). In the first column, (●) indicates specimens used for the species delimitation analyses and (○) indicates the specimens used for the multigene phylogenetic analyses.

	Taxon	Collector	Locality	Latitude	Longitude	Id number	Accession no.
○	*Canistrocarpus cervicornis*	Tronholm A., Sansón M., Afonso-Carrillo J.	Spain: Canary Islands: Tenerife: Punta Hidalgo	28.578	-16.328	D192	(See Table S2)
○	*Canistrocarpus cervicornis*	Verbruggen H.	Philippines: Bohol: Panglao	9.544	123.754	HV711	DQ472047
○	*Canistrocarpus cervicornis*	Verbruggen H., Van Nieuwenhuyze K.	Tanzania: Zanzibar: Matemwe	-5.889	39.361	TZ0714C	GQ466069
○	*Canistrocarpus crispatus*	De Clerck O.	Kenya: Tiwi	-4.245	39.604	ODC1545	GU265787
○	*Canistrocarpus crispatus*	Verbruggen H.	Philippines: Bohol: Panglao	9.544	123.759	HV721	(See Table S2)
○	*Canistrocarpus crispatus*	De Clerck O., Leliaert F., Payo D.A.	Philippines: Negros Oriental: Dumaguete	9.331	123.309	ODC1444	(See Table S2)
○	*Dictyopteris delicatula*	Tronholm A., Steen F.	Cape Verde: Sao Tiago: Pedra Badejo	15,142	-23,527	FS851	JQ061090
○	*Dictyopteris delicatula*	(See Bittner et al. 2008)					EU579943
○	*Dictyopteris divaricata*	(See Bittner et al. 2008)					AY422676
○	*Dictyopteris divaricata*	GenBank					AY430343
○	*Dictyopteris latiuscula*	(See Bittner et al. 2008)					AY422677
○	*Dictyopteris latiuscula*	GenBank					AY430349
○	*Dictyopteris polypodioides*	(See Silberfeld et al. 2010)					EU681404
○	*Dictyopteris polypodioides*	(See Silberfeld et al. 2010)					EU681445
○	*Dictyopteris polypodioides*	(See Bittner et al. 2008)					EU579932
○	*Dictyopteris polypodioides*	(See Silberfeld et al. 2010)					EU681639
○	*Dictyopteris polypodioides*	De Clerck O.	France: Marseille: Ile de Frioul	43.273	5.304	ODC1031	DQ472097
○	*Dictyopteris prolifera*	(See Bittner et al. 2008)					AY422678
○	*Dictyopteris prolifera*	GenBank					AY430346
○	*Dictyopteris undulata*	(See Bittner et al. 2008)					AY430333
○	*Dictyopteris undulata*	GenBank					AY430353
○ ●	*Dictyota acutiloba*	De Clerck O.	United States: Hawaii: Oahu: Honolulu: Ala Moana	21.286	-157.849	ODC888	(See Table S2)
○ ●	*Dictyota adnata*	Draisma S.G.A.	Indonesia: Raja Ampat: Gam	-0.519	130.570	SD712204	JQ061005 (See Table S2)
●	*Dictyota adnata*	Draisma S.G.A.	Indonesia: Raja Ampat: Gam: Besir Bay	-0.490	130.593	SD712293	JQ061006
○ ●	*Dictyota adnata*	De Clerck O, Leliaert F, Payo D.A.	Philippines: Negros Oriental: Bais	9.576	123.159	ODC1485	(See Table S2)

	Taxon	Collector	Locality	Latitude	Longitude	Id number	Accession no.
○ ●	*Dictyota bartayresiana*	De Clerck O.	Kenya: Diani Beach	-4.298	39.589	ODC1513	GQ425183
○ ●	*Dictyota bartayresiana*	De Clerck O.	Kenya: Kinondo Reef	-4.401	39.587	ODC1588	GQ425107
○ ●	*Dictyota bartayresiana*	Verbruggen H., Tyberghein L.	Tanzania: Zanzibar: Chwaka	-6.168	39.443	TZ0802C	GQ466071
○ ●	*Dictyota bartayresiana*	Tronholm B.	Dominican Republic: Punta Cana: Playa Bávaro	18.727	-68.460	DR7	(See Table S2)
●	*Dictyota bartayresiana*	Tronholm B.	Dominican Republic: Catalina Island	18.359	-69.023	DR16	JQ061009
●	*Dictyota bartayresiana*	Tronholm B.	Dominican Republic: Catalina Island	18.359	-69.023	DR17	JQ061010
●	*Dictyota bartayresiana*	Alfonso Y.	Cuba: Pinar del Río Province: Cabañas	22.989	-82.928	D627	JQ061007
●	*Dictyota bartayresiana*	De Clerck O.	Kenya: Chale Island	-4.450	39.533	ODC1663	JQ061008
●	*Dictyota bartayresiana*	Pauly K.	Tanzania: Mtwara: Ruvula	-10.317	40.398	TZ0277	JQ061013
●	*Dictyota bartayresiana*	Pauly K.	Tanzania: Mtwara: Ruvula	-10.317	40.398	TZ0279	JQ061014
○	*Dictyota binghamiae*	Lane C.E.	Canada: British Columbia (see Phillips et al. 2008)			Lane	EF990193
○ ●	*Dictyota binghamiae*	Verbruggen H., Zechman F.	Mexico: Baja California: La Bufadora	31.725	-116.719	HV1542	JQ061015
●	*Dictyota binghamiae*	Verbruggen H., Hernández J.	Baja California: Kennedy Camp	31.701	-116.683	HV1801	JQ061016
○ ●	*Dictyota binghamiae*	Verbruggen H., Hernández J.	Baja California: Kennedy Camp	31.701	-116.683	HV1802	JQ061017
●	*Dictyota canaliculata*	Draisma S.G.A.	Indonesia: Raja Ampat: Gam: Desa Besir	-0.463	130.687	SD712400	GQ466072
○	*Dictyota canaliculata*	Draisma S.G.A.	Indonesia: Raja Ampat: Yeffam Island	-0.569	130.274	SD712709	(See Table S2)
○ ●	*Dictyota canaliculata*	De Clerck O, Leliaert F, Payo D.A.	Philippines: Siquijor: Dapdap	9.224	123.516	ODC1477	(See Table S2)
○ ●	*Dictyota* cf. *caribaea*	Verbruggen H.	Jamaica: St. Ann Parish: St. Ann's Bay: Drax Hall	18.443	-77.182	HV926	(See Table S2)
●	*Dictyota* cf. *caribaea*	Moreira L.	Cuba: Cienfuegos Province: Bahia de Cienfuegos: Punta Gorda	22.133	-80.464	D630	JQ061058
●	*Dictyota* cf. *caribaea*	Cabrera R.	Cuba: Camaguey Province: Santa Cruz del Sur	20.697	-77.998	RC175	JQ061059
○ ●	*Dictyota ceylanica*	Verbruggen H.	French Polynesia: Tahiti: Faaa	-17.549	-149.618	HV214a	(See Table S2)
○ ●	*Dictyota ceylanica*	De Clerck O, Leliaert F, Payo D.A.	Philippines: Negros Oriental: Dumaguete	9.330	123.309	ODC1442	(See Table S2)
○ ●	*Dictyota ciliolata*	Tronholm A., Sansón M., Afonso-Carrillo J.	Spain: Canary Islands: Tenerife: Punta Hidalgo	28.578	-16.328	D191	(See Table S2)
○ ●	*Dictyota ciliolata*	Tronholm A.	Spain: Canary Islands: Gran Canaria: Faro de Maspalomas	27.734	-15.599	D395	GQ425192
○ ●	*Dictyota ciliolata*	Verbruggen H.	Philippines: Olango Island	10.215	123.997	HV632	GQ425124
●	*Dictyota ciliolata*	Verbruggen H.	Philippines: Bohol: Panglao	9.5435	123.759	HV723	GQ466073
●	*Dictyota ciliolata*	Breeman A.	Tenerife: Punta del Hidalgo	28.580	-16.325	LB2819	JQ061021
●	*Dictyota ciliolata*	De Clerck O, Leliaert F, Payo D.A.	Philippines: Negros Oriental: Dumaguete	9.331	123.309	ODC1441	JQ061022
●	*Dictyota ciliolata*	De Clerck O, Leliaert F, Payo D.A.	Philippines: Siquijor: Sawang	9.197	123.447	ODC1457	JQ061023

	Taxon	Collector	Locality	Latitude	Longitude	Id number	Accession no.
●	*Dictyota ciliolata*	De Clerck O.	Kenya: Kinondo Reef	-4.401	39.587	ODC1563	JQ061024
●	*Dictyota ciliolata*	Draisma S.G.A.	Indonesia: Raja Ampat: Gam	-0.536	130.579	SD712150	JQ061025
●	*Dictyota ciliolata*	Draisma S.G.A.	Indonesia: Raja Ampat: Gam	-0.536	130.579	SD712571	JQ061026
●	*Dictyota ciliolata*	Verbruggen H., Pauly K.	Tanzania: Mbudya Island	-6.653	39.248	TZ0046	JQ061027
●	*Dictyota ciliolata*	Pauly K.	Tanzania: Mtwara: Ruvula	-10.317	40.398	TZ0276	JQ061028
●	*Dictyota ciliolata*	Verbruggen H., Van Nieuwenhuyze K.	Tanzania: Zanzibar: Matemwe	-5.888	39.361	TZ0733	JQ061029
●	*Dictyota ciliolata* (as *D. menstrualis*)	Searles R.	United States: North Carolina: Beaufort: Radio Island	34.715	-76.691	Searles-2	JQ061064
○ ●	*Dictyota ciliolata* (as *D. plectens*)	Cowling T.	Australia: Queensland: Yeppoon: Keppel Bay	-23.136	150.760	TC2	JQ061075
○ ●	*Dictyota ciliolata* (as *D. plectens*)	Saunders G.W.	Australia: Lord Howe Island: Ned's Beach	159.066	-31.5167	GWS1029	JQ061074
●	*Dictyota coriacea*	Verbruggen H., Hernandez J.	Mexico: Baja California: Bahia Todos Santos: La Joya	31.719	-116.668	HV1810	JQ061030
○ ●	*Dictyota coriacea*	Murray S.	United States: California: Dana Point	33.459	-117.718	CSUF003	(See Table S2)
●	*Dictyota coriacea*	Lee W.J.	South Korea: Yeonam Gangwondo	36.037	129.436	WJ1	AY422613
○ ●	*Dictyota coriacea*	Lee W.J.	South Korea: Yeonam Gangwondo	36.037	129.436	WJ3	AY422612
○ ●	*Dictyota crenulata* #1	Verbruggen H.	Mexico: Baja California Sur: Cabo San Lucas: Playa Santa Maria	22.930	-109.815	HV1074	(See Table S2)
●	*Dictyota crenulata* #1	Verbruggen H., Tyberghein L.	Mexico: Oaxaca: Mazunte: Mazunte Beach	15.660	-96.555	MX0082	JQ061032
●	*Dictyota crenulata* #1	Fernández C.	Mexico: Oaxaca: Bahia de Huatulco: Togolunda	15,665	-96,496	CFMX206	JQ061089
○ ●	*Dictyota crenulata* #2	Tronholm A.	Spain: Canary Islands: Tenerife: Playa de Las Arenas	28.371	-16.877	D319	(See Table S2)
○ ●	*Dictyota crenulata* #2	Tronholm A.	Spain: Canary Islands: Tenerife: Playa de Las Arenas	28.371	-16.877	D504	(See Table S2)
●	*Dictyota crenulata* #2	Tronholm A., Steen F.	Cape Verde: Sal: Pedra Lume	16.762	-22.889	FS927	JQ061084
●	*Dictyota crenulata* #2	Tronholm A., Steen F.	Cape Verde: Santo Antao: Ponta do Sol	17.2032	-25.094	FS987	JQ061085
●	*Dictyota crenulata* #2	Tronholm A., Steen F.	Cape Verde: Sal: Baia da Murdeira	16.678	-22.937	FS879	JQ061086
●	*Dictyota crenulata* #2	Tronholm A., Steen F.	Cape Verde: Sal: Baia da Murdeira	16.678	-22.937	FS871	JQ061087
●	*Dictyota crenulata* #2	Tronholm A., Steen F.	Cape Verde: Sal: Pedra Lume	16.762	-22.889	FS928	JQ061088
○ ●	*Dictyota crenulata* #3	Tronholm B.	Dominican Republic: Punta Cana: Playa Bávaro	18.727	-68.460	DR27	JQ061055 (See Table S2)
●	*Dictyota crenulata* #3	Tronholm B.	Dominican Republic: Punta Cana: Playa Bávaro	18.727	-68.460	DR28	JQ061056
●	*Dictyota crenulata* #3	Alfonso Y.	Cuba: Habana: Miramar	23.134	-82.413	D631	JQ061054
●	*Dictyota crenulata* #3	Cabrera R.	Cuba: Miramar: Calle 42	23.120	-82.434	RC154	JQ061057

	Taxon	Collector	Locality	Latitude	Longitude	Id number	Accession no.
●	*Dictyota crenulata* #3	Tronholm A., Steen F.	Cape Verde Islands: Sal: Baia da Murdeira	16,678	-22,935	FS881	JQ061083
○ ●	*Dictyota crenulata* #4	Tronholm A.	Spain: Canary Islands: Tenerife: Punta Hidalgo	28.580	-16.325	D193	JQ061093
○ ●	*Dictyota crenulata* #4	Tronholm A.	Spain: Canary Islands: Tenerife: Buenavista: Playa de Las Arenas	28.371	-16.871	D324	JQ061050 (See Table S2)
○ ●	*Dictyota crenulata* #4	Tronholm A.	Spain: Canary Islands: Gran Canaria: Faro de Maspalomas	27.734	-15.599	D394	JQ061051
○	*Dictyota crenulata* #4	Tronholm A.	Spain: Canary Islands: Tenerife: El Médano	28.044	-16.536	D404	(See Table S2)
●	*Dictyota crenulata* #4	Tronholm A.	Spain: Canary Islands: Tenerife: Punta Hidalgo	28.580	-16.325	D310	JQ061049
●	*Dictyota crenulata* #4	Tronholm A.	Spain: Canary Islands: Tenerife: Punta Hidalgo	28.580	-16.325	D400	JQ061052
●	*Dictyota crenulata* #4	Tronholm A.	Spain: Canary Islands: Tenerife: Punta Hidalgo	28.580	-16.325	D401	JQ061053
○ ●	*Dictyota cyanoloma*	Tronholm A.	Spain: Canary Islands: Gran Canaria: Arinaga: Zoco del Negro	27.866	-15.384	D502	(See Table S2)
○ ●	*Dictyota cyanoloma*	Tronholm A.	Portugal: Algarve: Portimão: Praia da Rocha	37.117	-8.547	D544	(See Table S2)
●	*Dictyota cyanoloma*	Lluch R.	Spain: Barcelona	41.331	2.173	D621	GU255592
●	*Dictyota cyanoloma*	Lluch R.	Spain: Barcelona	41.331	2.173	D623	GU255593
●	*Dictyota cyanoloma*	Pauly K.	Portugal: Algarve: Carvoeiro: A Boneca	37.093	-8.465	FAO006	GU255594
●	*Dictyota cyanoloma*	Steen F.	Portugal: Algarve: Lagos: Praia da Dona Ana	37.091	-8.669	FS539	JQ061033
●	*Dictyota cyanoloma*	Steen F.	Portugal: Algarve: Porto Covo	37.827	-8.792	FS428	JQ061034
●	*Dictyota cyanoloma*	Steen F.	Portugal: Algarve: Porto Covo	37.827	-8.792	FS429	JQ061035
●	*Dictyota cyanoloma*	Steen F.	Portugal: Algarve:Albufeira: Olhos de Agua	37.089	-8.194	FS469	JQ061036
●	*Dictyota cyanoloma*	Coppejans E., De Clerck O.	Portugal: Madeira Island: Rais Magos	32.602	-16.804	HEC15777	GU255595
●	*Dictyota cyanoloma*	Amaral, Alvaro, Couto	Portugal: Azores: Pico: Santa Barbara	38.391	-28.213	Pix07637	GU255544
○ ●	*Dictyota cymatophila*	Tronholm A.	Spain: Canary Islands: Tenerife: Punta del Hidalgo	28.580	-16.325	D306	GQ425193
○ ●	*Dictyota cymatophila*	Tronholm A.	Spain: Canary Islands: Gran Canaria: El Berriel	27.785	-15.501	D397	GQ425197 (See Table S2)
○ ●	*Dictyota cymatophila*	Tronholm A.	Spain: Canary Islands: Tenerife: Punta del Hidalgo	28.580	-16.325	D403	GQ425199 (See Table S2)
○ ●	*Dictyota cymatophila*	Tronholm A.	Spain: Canary Islands: Tenerife: El Médano	28.044	-16.536	D406	GQ425201 (See Table S2)
●	*Dictyota cymatophila*	Tronholm A.	Spain: Canary Islands: Tenerife: Punta del Hidalgo	28.580	-16.325	D399	GQ425198
●	*Dictyota cymatophila*	Tronholm A.	Spain: Canary Islands: Gran Canaria: Faro de Maspalomas	27.734	-15.599	D393	GQ425196
●	*Dictyota cymatophila*	Tronholm A.	Spain: Canary Islands: Gran Canaria: Faro de Maspalomas	27.734	-15.599	D392	GQ425195
●	*Dictyota cymatophila*	Tronholm A.	Spain: Canary Islands: Tenerife: El Médano	28.044	-16.536	D405	GQ425200

	Taxon	Collector	Locality	Latitude	Longitude	Id number	Accession no.
○ ●	*Dictyota dhofarensis*	Schils T., Pauly K., Provoost P.	Oman: Dhofar: Mirbat	17.050	55.086	DHO0163	(See Table S2)
○ ●	*Dictyota dichotoma*	Tronholm A., Sansón M., Afonso-Carrillo J.	Spain: Canary Islands: Tenerife: Punta Hidalgo	28.578	-16.328	D190	GQ425202 (See Table S2)
●	*Dictyota dichotoma*	Tronholm A.	Spain: Canary Islands: Tenerife: Abades	28.136	-16.442	ATV78	GU255618
●	*Dictyota dichotoma*	Tronholm A.	Spain: Canary Islands: Tenerife: Punta de Teno	28.342	-16.924	ATV101	GU255637
○	*Dictyota dichotoma*	De Clerck O.	France: Nord-Pas-de-Calais: Audreselles: Pointe du Nid de Corbet	50.829	1.590	ODC1027	DQ472051
○	*Dictyota dichotoma*	De Clerck O.	France: Languedoc-Roussillon: Banyuls: Cap du Troc	42.481	3.143	ODC1055	GQ425131
●	*Dictyota dichotoma*	De Clerck O., Rodriguez-Prieto C.	Spain: Cataluña: Girona: Begur	41.961	3.230	ODC1688	GU255541
○	*Dictyota dichotoma*	De Clerck O., Zuccarello G.C.	United Kingdom: England: Barrow-in-furness: Walney Island	54.080	-3.245	ODC1689	GU255542
●	*Dictyota dichotoma*	De Clerck O. & Rodriguez-Prieto C.	Spain : Cataluña: Begur: Sa Tuna	41.961	3.230	ODC1695	GU255543
●	*Dictyota dichotoma*	Pauly K.	Portugal: Algarve: Carvoeiro: A Boneca	37.093	-8.465	FAO005	GU255530
●	*Dictyota dichotoma*	Kooistra W.	Italy: Naples: Posilipo	40.794	14.1934	Kooistra2	GU255536
●	*Dictyota dichotoma*	Steen F., De Clerck O.	France: Brittany: Roscoff: Ile Verte	48.728	-3.984	FS094	GU255774
●	*Dictyota dichotoma*	Steen F.	France: Brittany: Pointe de Raz: Anse de Loc'h	48.029	-4.635	FS114	GU255809
●	*Dictyota dichotoma*	Steen F., Hollants J.	France: Brittany: Pointe de Mousterlin	47.845	-4.012	FS160	FJ869842
●	*Dictyota dichotoma*	Steen F., Hollants J., Verlaque M.	France: Provence: Carry-le-Rouet: Sausset les Pins	43.329	5.105	FS255	GU255778
●	*Dictyota dichotoma*	Steen F., Hollants J., Verlaque M.	France: Provence: Cassis: Cap Canaille	43.207	5.547	FS327	GU255795
●	*Dictyota dichotoma*	Poloniato D.	Italy: Trieste	45.605	13.713	DP001	GU255769
●	*Dictyota dichotoma*	Coppejans E.	France: Bretagne: Roscoff: Ile Verte	48.732	-3.989	HEC15604	GU255531
●	*Dictyota dichotoma*	Azevedo, Torrão	Portugal: Azores: Pico: Manhenha	38.411	-28.033	Pix071332	GU255765
●	*Dictyota dichotoma*		Ireland: Galway				AY528442
●	*Dictyota fasciola*	De Clerck O.	France: Languedoc-Roussillon: Port Vendres: Les Paulilles	42.522	3.114	ODC1045	GU255551
○ ●	*Dictyota fasciola*	De Clerck O.	France: Languedoc-Roussillon: Banyuls: Cap du Troc	42.481	3.143	ODC1057	FJ869846 (See Table S2)
○ ●	*Dictyota fasciola*	De Clerck O.	France: Languedoc-Roussillon: Côte Vermeille: Cap Peyferite	42.454	3.166	ODC1065	GQ466074 (See Table S2)
○	*Dictyota fasciola*	De Clerck O.	France: Languedoc-Roussillon: Côte Vermeille: Cap Peyferite	42.454	3.166	ODC1066	FJ869847
●	*Dictyota fasciola*	Tronholm A.	Spain: Canary Islands: Tenerife: Playa de Las Arenas	28.371	-16.872	ATV94	GU255633
●	*Dictyota fasciola*	Tronholm A.	Spain: Canary Islands: Lanzarote: Famara	29.119	-13.564	D271	GQ425209
●	*Dictyota fasciola*	Tronholm A.	Spain: Canary Islands: Tenerife: Punta del Hidalgo	28.580	-16.325	D307	GU255546

	Taxon	Collector	Locality	Latitude	Longitude	Id number	Accession no.
●	*Dictyota fasciola*	Tronholm A., Sansón M., Afonso-Carrillo J.	Spain: Canary Islands: Tenerife: El Médano	28.044	-16.536	D344	GQ425211
●	*Dictyota fasciola*	Tronholm A.	Spain: Canary Islands: Gran Canaria: Medio Almud	27.800	-15.739	D354	GQ425212
●	*Dictyota fasciola*	Furnari G.	Italy: Siracusa: Porto Palo	36.689	15.138	D643	GU255548
○ ●	*Dictyota friabilis*	Verbruggen H., Zubia M.	French Polynesia: Tahiti: Arue	-17.520	-149.547	HV153	(See Table 2)
○ ●	*Dictyota friabilis*	Littler D., Littler M.	Netherlands Antilles: Saba: Saba Bank	17.508	-63.440	DML67250	JQ061038 (See Table 2)
○ ●	*Dictyota friabilis*	De Clerck O.	United States: Hawaii: Oahu: Lanikai	21.396	-157.720	ODC898	JQ061041 (See Table 2)
●	*Dictyota friabilis*	De Clerck O, Leliaert F, Payo D.A.	Philippines: Negros Oriental: Apo Island	9.083	123.25	ODC1492	JQ061040
●	*Dictyota friabilis*	Draisma S.G.A.	Indonesia: Raja Ampat: Sleeping Barracuda reef	-0.545	130.7	SD712192	JQ061042
○ ●	*Dictyota grossedentata*	Pauly K.	Tanzania: Zanzibar: Mnemba atoll	-5.838	39.392	TZ0490	(See Table 2)
○ ●	*Dictyota hamifera*	Verbruggen H., N'Yeurt A.	French Polynesia: Tahiti: Afaahiti	-17.738	-149.262	HV222	(See Table 2)
○	*Dictyota hamifera*	Littler D., Littler M.	Netherlands Antilles: Saba: Saba Bank	17.508	-63.441	DML67438	GQ425112
○ ●	*Dictyota humifusa*	De Clerck O.	Kenya: Chale Island	-4.450	39.533	ODC1659	JQ061044 (See Table 2)
○ ●	*Dictyota humifusa*	Draisma S.G.A.	Indonesia: Raja Ampat: Kri	-0.556	130.691	SD712066	JQ061045 (See Table 2)
○ ●	*Dictyota humifusa*	Draisma S.G.A.	Indonesia: Raja Ampat: Gam	-0.429	130.56	SD712460	(See Table 2)
○ ●	*Dictyota implexa*	De Clerck O., Heytens M.	Spain: Cataluña: Girona: Palamós: Cala Corbs	41.858	3.147	ODC1238	(See Table 2)
○	*Dictyota implexa*	Steen F., Hollants J., Verlaque M.	Provence: Carry-le-Rouet: Sausset les Pins	43.329	5.105	FS271	GQ425135
○	*Dictyota implexa*	Le Gall L.	Croatia: Mljet Island: Prozura	42.730	17.653	LLGO249	GQ425140
○	*Dictyota implexa*	Le Gall L.	Croatia: Korcula Island: Badija	42.951	17.163	LLGO300	GQ425168
○ ●	*Dictyota implexa*	Kooistra W.	Italy: Naples: Posilipo	40.794	14.193	Kooistra1	GU255588 (See Table S2)
●	*Dictyota implexa*	Pena C.	Spain: Baleares: Menorca: Cap Morell	40.055	3.879	ABH599	GU255574
●	*Dictyota implexa*	Tronholm A.	Spain: Canary Islands: Tenerife: Playa de Las Arenas	28.371	-16.872	ATV93	GU255632
●	*Dictyota implexa*	Lane C.E, Schneider C.W.	Bermuda: Saint George	32.357	-64.683	CLO30101	GQ425216
●	*Dictyota implexa*	Tronholm A.	Spain: Canary Islands: Lanzarote: Puerto del Carmen	28.919	-13.666	D282	GU255575
●	*Dictyota implexa*	Tronholm A.	Spain: Canary Islands: La Graciosa: Caleta del Sebo	29.225	-13.505	D288	GU255577
●	*Dictyota implexa*	Tronholm A.	Spain: Canary Islands: Tenerife: La Guancha: Charco del Viento	28.401	-16.673	D303	GU255579
●	*Dictyota implexa*	Tronholm A.	Spain: Canary Islands: Tenerife: Playa de Las Arenas	28.371	-16.872	D327	GU255580

	Taxon	Collector	Locality	Latitude	Longitude	Id number	Accession no.
●	*Dictyota implexa*	Tronholm A.	Spain: Canary Islands: Gran Canaria: Medio Almud	27.800	-15.739	D349	GU255581
●	*Dictyota implexa*	Tronholm A.	Spain: Canary Islands: Gran Canaria: Arinaga: Zoco del Negro	27.866	-15.385	D506	GU255584
●	*Dictyota implexa*	Poloniato D.	Italy: Trieste	45.605	13.713	DP002	GU255770
●	*Dictyota implexa*	De Clerck O.	France: Languedoc-Roussillon: Côte Vermeille: Cerbère: Cap Peyferite	42.454	3.166	ODC1073	GU255589
●	*Dictyota implexa*	Azevedo, Couto, Torrão	Portugal: Azores: Pico: Feteira	38.402	-28.068	PIX071434	GU255766
●	*Dictyota implexa*	Bárbara I.	Algarve: Armacao de Pera	37.100	-8.358	Sant19426	GU255583
○ ●	*Dictyota intermedia*	Cowling T.	Australia: Queensland: Yeppoon: Keppel Bay	-23.136	150.760	TC1	(See Table S2)
●	*Dictyota intermedia*	Saunders G.W.	Australia: Lord Howe Island	-31.552	159.081	GWS1020	JQ061047
○ ●	*Dictyota kunthii*	Faugeron S.	Chile: Pan de Azúcar	-26.151	-70.670	D102	(See Table S2)
○	*Dictyota kunthii*	Faugeron S.	Chile: Maitencillo	-31.470	-71.588	D104	GU290231
○ ●	*Dictyota liturata*	Coppejans E., De Clerck O.	Portugal: Madeira Island: Rais Magos	32.602	-16.804	HEC15721	JQ061063 (See Table S2)
○ ●	*Dictyota liturata*	Coppejans E., De Clerck O.	Portugal: Madeira Island: Porto da Cruz	32.773	-16.827	HEC15816	GQ466075
○ ●	*Dictyota liturata*	De Clerck O., Leliaert F.	South Africa: Durban: The Bluff	-34.120	18.460	KZN2282	GQ425214 (See Table S2)
○	*Dictyota liturata*	Solé M.	Venezuela: Isla De Margarita: Porlamar: Playa El Agua	10.953	-63.844	Sole1	GQ425113
●	*Dictyota liturata*	Tronholm A.	Portugal: Madeira Island: Rais Magos	32.602	-16.804	D693	JQ061060
●	*Dictyota liturata*	Tronholm A.	Portugal: Madeira Island: Rais Magos	32.602	-16.804	D694	JQ061061
●	*Dictyota liturata*	Coppejans E., De Clerck O.	Portugal: Madeira: Funchal: Cais de Carvão	32.634	-16.940	HEC15674	JQ061062
○	*Dictyota mediterranea*	Le Gall L.	Croatia: Hvar Island	43.155	16.387	LLGO224	GU290246
○ ●	*Dictyota mediterranea*	Le Gall L.	Croatia: Korcula Island: Kneza	42.971	17.048	LLGO313	GU255570 (See Table S2)
○ ●	*Dictyota mediterranea*	Furnari G.	Italy: Siracusa: Porto Palo	36.689	15.138	D653	GU255569
○	*Dictyota mediterranea*	Draisma S.G.A.	Italy: Sicily: Giardini Naxos	37.822	15.278	SGAD1116	(See Table S2)
○	*Dictyota mediterranea*	Pena C., Martínez M., Crespo M.B.	Spain: Baleares: Mallorca	39.788	3.178	D595	GU290254
●	*Dictyota mediterranea*	Furnari G.	Italy: Agrigento: Siculiana	37.314	13.414	D633	GU255566
●	*Dictyota mediterranea*	Furnari G.	Italy: Siracusa: Porto Palo	36.689	15.138	D651	GU255568
○	*Dictyota mertensii*	Verbruggen H.	Jamaica: St. Ann Parish: Priory	18.456	-77.226	HV911	GQ425158
○ ●	*Dictyota mertensii*	Tronholm B.	Dominican Republic: Punta Cana: Playa Bávaro	18.727	-68.460	DR31	(See Table S2)

	Taxon	Collector	Locality	Latitude	Longitude	Id number	Accession no.
○ ●	*Dictyota mertensii*	Tronholm B.	Dominican Republic: Punta Cana: Playa Bávaro	18.727	-68.460	DR32	JQ061065 (See Table S2)
○ ●	*Dictyota naevosa*	De Clerck O., Schils T., Verbruggen H., Demeulenaere E.	South Africa: Kwazulu-Natal: Palm Beach	-31.003	30.2670	KZNb2345	JQ061067 (See Table S2)
○ ●	*Dictyota naevosa*	De Clerck O., Leliaert F.	South Africa: Kwazulu-Natal: Mission Rocks	-28.277	32.506	KZN2241	(See Table S2)
●	*Dictyota naevosa*	Anderson R.J.	South Africa: Western Cape Province: De Hoop Nature Reserve	-34.498	20.477	D659	JQ061066
○ ●	*Dictyota nigricans*	Huisman J.	Australia: Western Australia: Perth: Penguin Island	-32.311	115.691	D92	(See Table S2)
○	*Dictyota pinnatifida*	Lane C., Schneider C.W.	Bermuda: Horseshoe Bay	32.252	-64.821	CLO31302	GQ425157
○ ●	*Dictyota pinnatifida*	Verbruggen H.	Jamaica: St. Ann Parish: Priory	18.449	-77.217	HV902	(See Table S2)
○	*Dictyota pinnatifida*	Verbruggen H.	Jamaica: St. Ann Parish: Priory: Chris Cove	18.456	-77.233	HV932	(See Table S2)
○ ●	*Dictyota pinnatifida*	Solé M.	Venezuela: Isla De Margarita: Playa El Tirano	11.119	-63.842	Sole3	JQ061073 (See Table S2)
●	*Dictyota pinnatifida*	Tronholm B.	Dominican Republic: Punta Cana: Playa Bávaro	18.727	-68.460	DR1	JQ061069
●	*Dictyota pinnatifida*	Tronholm B.	Dominican Republic: Punta Cana: Playa Bávaro	18.727	-68.460	DR4	JQ061070
●	*Dictyota pinnatifida*	Tronholm B.	Dominican Republic: Punta Cana: Playa Bávaro	18.727	-68.460	DR5	JQ061071
●	*Dictyota pinnatifida*	Tronholm B.	Dominican Republic: Punta Cana: Playa Bávaro	18.727	-68.460	DR6	JQ061072
○ ●	*Dictyota rigida*	De Clerck O.	Kenya: Mombasa: McKenzie Point	-4.067	39.689	ODC1623	JQ061077 (See Table S2)
○ ●	*Dictyota rigida*	De Clerck O.	Kenya: Mombasa: McKenzie Point	-4.067	39.689	ODC1657	(See Table S2)
●	*Dictyota rigida*	De Clerck O.	Kenya: Tiwi	-4.245	39.604	ODC1553	JQ061076
●	*Dictyota rigida*	Verbruggen H., Van Nieuwenhuyze K.	Tanzania: Zanzibar: Matemwe	-5.888	39.361	TZ0731C	GQ425217
○ ●	*Dictyota sandvicensis*	De Clerck O.	United States: Hawaii: Oahu: Honolulu: Ala Moana	21.286	-157.849	ODC889	(See Table S2)
○ ●	*Dictyota sandvicensis*	De Clerck O.	United States: Hawaii: Oahu: Lanikai	21.396	-157.720	ODC896	(See Table S2)
○ ●	*Dictyota spiralis*	De Clerck O.	France: Provence-Alpes-Côte d'Azur: Île de Frioul	43.277	5.303	ODC1029	GQ425221 (See Table S2)
○ ●	*Dictyota spiralis*	De Clerck O.	France: Languedoc-Roussillon: Côte Vermeille: Cerbère: Cap Peyferite	42.454	3.166	ODC1071	GU255606 (See Table S2)
○ ●	*Dictyota spiralis*	Coppejans E., De Clerck O.	Portugal: Madeira Island: Porto da Cruz	32.773	-16.827	HEC15815	GQ466078
○	*Dictyota spiralis*	De Clerck O., Heytens M.	Spain: Cataluña: Girona: Palamós: Cala Corbs	41.858	3.147	ODC1225	GQ425161
●	*Dictyota spiralis*	Pena C.	Spain: Baleares: Menorca: Son Bou	39.899	4.072	ABH596	GU255596
●	*Dictyota spiralis*	Tronholm A.	Spain: Canary Islands: Tenerife: Punta de Teno	28.342	-16.924	ATV103	GU255638

	Taxon	Collector	Locality	Latitude	Longitude	Id number	Accession no.
●	*Dictyota spiralis*	Tronholm A., Sansón M., Afonso-Carrillo J.	Spain: Canary Islands: Tenerife: El Médano	28.0436	-16.536	D343	GQ425219
●	*Dictyota spiralis*	Tronholm A.	Spain: Canary Islands: Tenerife: Punta Hidalgo	28.580	-16.325	D308	GU255597
●	*Dictyota spiralis*	Tronholm A.	Portugal: Algarve: Albufeira: Galé	37.078	-8.312	D543	GU255598
●	*Dictyota spiralis*	Steen F., De Clerck O.	France: Normandy: St. Enogat	48.641	-2.071	FS068	GU255839
●	*Dictyota spiralis*	Steen F., Hollants J., Verlaque M.	France: Provence: Carry-le-Rouet: Sausset les Pins	43.329	5.105	FS231	GU255836
●	*Dictyota spiralis*	Steen F., Hollants J., Verlaque M.	France: Provence: Cassis: Cap Canaille	43.207	5.547	FS301	FJ869838
●	*Dictyota spiralis*	Steen F.	Portugal: Algarve: Lagos: Praia da Dona Ana	37.091	-8.669	FS498	JQ061079
●	*Dictyota spiralis*	De Clerck O.	France: Languedoc-Roussillon: Banyuls: Cap du Troc	42.481	3.143	ODC1056	GU255604
●	*Dictyota spiralis*	Draisma S.G.A.	Italy: Sicily: Giardini Naxos	37.822	15.278	SGAD1115	GU255608
○ ●	*Dictyota stolonifera*	Verbruggen H., Díaz R., Galanza C.	Philippines: Luzon: Sorsogon: Bulusan: Dancalan	12.849	124.153	HV819	GQ425222
○	*Dictyota stolonifera*	Lin S.M.	Taiwan: Keelung City: Batouzi Harbor			D264	GQ425118
○ ●	*Dictyota stolonifera*	Pauly K.	Tanzania: Zanzibar: Mnemba atoll	-5.838	39.392	TZ0488	JQ061082 (See Table S2)
●	*Dictyota stolonifera*	Verbruggen H.	Egypt: Marsa Alam	25.069	34.904	HV1831	JQ061080
●	*Dictyota stolonifera*	Pauly K.	Tanzania: Mtwara: Mikindani Bay	-10.198	40.144	TZ0377	JQ061081
○	*Dilophus fastigiatus*	Goldberg N.	Australia: South Australia: Esperance Bay: Woody Island	-33.980	121.931	D96	(See Table S2)
○	*Padina arborescens*	GenBank					EU579996
○	*Padina arborescens*	(See Ni-Ni-Win et al. 2010)					AB358939
○	*Padina arborescens*	GenBank					AY430357
○	*Padina arborescens*	(See Ni-Ni-Win et al. 2010)					AB358904
○	*Padina boergesenii*	De Clerck O.	Hawaii: Oahu: Honolulu: Ala Moana	21.285	-157.850	ODC890	(See Table S2)
○	*Padina crassa*	(See Ni-Ni-Win et al. 2010)					AB358943
○	*Padina crassa*	GenBank					AY430361
○	*Padina japonica*	(See Ni-Ni-Win et al. 2010)					AB358942
○	*Padina japonica*	GenBank					AY430360
○	*Padina japonica*	(See Ni-Ni-Win et al. 2010)					AB358910
○	*Padina pavonica*	GenBank					EU580000
○	*Padina pavonica*	(See Silberfeld et al. 2010)					EU681454
○	*Padina pavonica*	(See Silberfeld et al. 2010)					EU681498
○	*Padina pavonica*	(See Silberfeld et al. 2010)					EU681649

	Taxon	Collector	Locality	Latitude	Longitude	Id number	Accession no.
○	*Padina pavonica*	(See Bittner et al. 2008)					EU579961
○	*Padina sanctae-crucis*	(See Ni-Ni-Win et al. 2010)					AB489969
○	*Padina sanctae-crucis*	Lane C.E, Schneider C.W.	Bermuda: St. George's Island: Whalebone Bay	32.365	-64.713	CL030305	(See Table S2)
○	*Rugulopteryx okamurae*	Steen F., Hollants J., Verlaque M.	France: Languedoc-Roussillon: Lagune de Thau: Mèze	43.421	3.607	FS280	(See Table S2)
○	*Rugulopteryx okamurae*	Hwang I.K.	South Korea: Haegeumgang	34.733	128.634	D194	(See Table S2)
○	*Scoresbyella profunda*	Huisman J.	Australia: Western Australia: Geographe Bay	-33.654	115.262	DIC44	(See Table S2)
○	*Spatoglossum crassum*	GenBank					AY430355
○	*Spatoglossum crassum*	GenBank					AY422679
○	*Spatoglossum asperum*	GenBank					EU580003
○	*Spatoglossum asperum*	GenBank					EU579964
