# Supplementary material for: Contrasting Geographical Distributions as a Result of Thermal Tolerance and Long-Distance Dispersal in Two Allegedly Widespread Tropical Brown Algae
Source: PLoS One. 2012 Jan 26;7(1):e30813. doi: 10.1371/journal.pone.0030813 (PMC3266907; doi:10.1371/journal.pone.0030813)
Supplement: Table S2 — Genbank accession numbers of the sequences used in the concatenated alignment, including strain numbers and sequence length. (PDF) [file pone.0030813.s004.pdf]

Table S2. Genbank accession numbers of the sequences used in the concatenated alignment, including strain numbers and sequence length.

Taxon	LSU	*cox*1	*cox*3	*nad*1	*psb*A	*rbc*L
Canistrocarpus cervicornis	JQ061091<br>D192<br>1175 nt			GQ425184<br>D192<br>717 nt	GQ466069<br>TZ0714C<br>871 nt	DQ472047<br>HV711<br>1258 nt
Canistrocarpus crispatus	GQ425150<br>HV721<br>1171 nt	GQ425137<br>ODC1444<br>610 nt	GQ425146<br>ODC1444<br>648 nt	GQ425176<br>ODC1444<br>717 nt	GU265787<br>ODC1545<br>870 nt	GQ425119<br>HV721<br>1220 nt
Dictyopteris delicatula					JQ061090<br>FS851<br>889nt	EU579943<br><br>1095nt
Dictyopteris divaricata					AY430343<br><br>870nt	AY422676<br><br>1292nt
Dictyopteris latiuscula					AY430349<br><br>870nt	AY422677<br><br>1292nt
Dictyopteris polypodioides	DQ472097<br>ODC1031<br>1272nt	EU681404<br><br>639nt	EU681445<br><br>656nt		EU681639<br><br>864nt	EU579932<br><br>1271nt
Dictyopteris prolifera					AY430346<br><br>870nt	AY422678<br><br>1292nt
Dictyopteris undulata					AY430353<br><br>870nt	AY430333<br><br>1292nt
Dictyota acutiloba	DQ472111<br>ODC888<br>1169 nt	GU290238<br>ODC888<br>605 nt	GU290242<br>ODC888<br>276 nt	GU290247<br>ODC888<br>717 nt	EU395602<br>ODC888<br>855 nt	DQ472056<br>ODC888<br>1162 nt
Dictyota adnata	GQ425154<br>ODC1485<br>1052 nt	GQ425134<br>SD712204<br>548 nt		GQ425178<br>ODC1485<br>709 nt	GU265788<br>SD712204<br>866 nt	GQ425106<br>SD712204<br>1234 nt
Dictyota bartayresiana	GQ425153<br>DR7<br>1098 nt	GQ425129<br>DR7<br>617 nt		GQ425183<br>ODC1513<br>717 nt	GQ466071<br>TZ0802C<br>873 nt	GQ425107<br>ODC1588<br>1280 nt
Dictyota binghamiae	EF990193<br>Lane<br>1174 nt				JQ061015<br>HV1542<br>861 nt	JQ061120<br>HV1801<br>1129 nt
Dictyota canaliculata	GQ425167<br>SD712709<br>1097 nt	GQ425132<br>ODC1477<br>642 nt		GQ425177<br>ODC1477<br>717 nt	GQ425190<br>ODC1477<br>824 nt	GQ425108<br>SD712709<br>1277 nt
Dictyota* cf. *caribaea	DQ472116<br>HV926<br>1157 nt	JQ061097<br>HV926<br>633 nt	JQ061107<br>HV926<br>653 nt	JQ061110<br>HV926<br>717 nt	EU395608<br>HV926<br>858 nt	DQ472061<br>HV926<br>1146 nt
Dictyota ceylanica	GQ425152<br>HV214a<br>1167 nt	GQ425122<br>HV214a<br>621 nt	GQ425145<br>ODC1442<br>657 nt	GQ425175<br>ODC1442<br>704 nt	EU395607<br>HV214a<br>858 nt	DQ472067<br>HV214a<br>1250 nt
Dictyota ciliolata	GQ425156<br>D191<br>1174 nt	GQ425124<br>HV632<br>626 nt		GQ425173<br>D191<br>717 nt	GQ425192<br>D395<br>871 nt	GQ425109<br>D191<br>1239 nt

Taxon	LSU	*cox*1	*cox*3	*nad*1	*psb*A	*rbc*L
Dictyota coriacea	DQ472109<br>CSUF003<br>1162 nt	GU290234<br>CSUF003<br>621 nt		GU290251<br>CSUF003<br>717 nt	AY422612<br>WJ3<br>882 nt	DQ472054<br>CSUF003<br>1223 nt
***Dictyota crenulata* #1**	GU290231<br>HV1074<br>999 nt			GU290252<br>HV1074<br>717 nt	GU265782<br>HV1074<br>871 nt	GU290253<br>HV1074<br>1271 nt
***Dictyota crenulata* #2**	JQ061092<br>D504<br>979 nt	JQ061098<br>D319<br>634 nt	JQ061108<br>D319<br>657 nt	JQ061111<br>D319<br>717 nt	JQ061018<br>D319<br>871 nt	JQ061121<br>D504<br>1270 nt
***Dictyota crenulata* #3**		JQ061099<br>DR27<br>633 nt		JQ061112<br>DR27<br>717 nt	JQ061055<br>DR27<br>859 nt	
***Dictyota crenulata* #4**	JQ061093<br>D193<br>1173 nt	JQ061100<br>D404<br>617 nt		JQ061113<br>D404<br>717 nt	JQ061051<br>D394<br>871 nt	JQ061122<br>D324<br>1199 nt
Dictyota cyanoloma	JQ061094<br>D502<br>1012 nt	JQ061101<br>D544<br>600 nt		JQ061114<br>D544<br>717 nt	GU255590<br>D502<br>871 nt	JQ061123<br>D544<br>1275 nt
Dictyota cymatophila	GQ425162<br>D397<br>1016 nt	GQ425128<br>D406<br>622 nt		GQ425179<br>D403<br>717 nt	GQ425193<br>D306<br>872 nt	GQ425111<br>D397<br>1135 nt
Dictyota dhofarensis	DQ472127<br>DHO0163<br>1174 nt	JQ061102<br>DHO0163<br>603 nt		JQ061115<br>DHO0163<br>717 nt	JQ061037<br>DHO0163<br>752 nt	JQ061124<br>DHO0163<br>782 nt
Dictyota dichotoma	GQ425155<br>D190<br>1174 nt	GQ425131<br>ODC1055<br>645 nt	AY500368<br>GenBank<br>657 nt	AY500368<br>Genbank<br>717 nt	GU255542<br>ODC1689<br>882 nt	DQ472051<br>ODC1027<br>1262 nt
Dictyota fasciola	GQ425166<br>ODC1057<br>1137 nt	GQ425133<br>ODC1065<br>641 nt	GQ425143<br>ODC1065<br>654 nt	GQ425172<br>ODC1065<br>717 nt	FJ869847<br>ODC1066<br>871 nt	GQ425110<br>ODC1065<br>1287 nt
Dictyota friabilis	DQ472120<br>ODC898<br>1171 nt	GU290237<br>DML67250<br>536 nt	GU290244<br>ODC898<br>655 nt	GU290249<br>ODC898<br>717 nt	GU265786<br>HV153<br>861 nt	DQ472064<br>HV153<br>1250 nt
Dictyota grossedentata	JQ061095<br>TZ0490<br>846 nt	JQ061103<br>TZ0490<br>641 nt		JQ061116<br>TZ0490<br>717 nt	JQ061043<br>TZ0490<br>861 nt	JQ061125<br>TZ0490<br>1293 nt
Dictyota hamifera	DQ472110<br>HV222<br>1171 nt	GQ425123<br>HV222<br>618 nt	GQ425141<br>HV222<br>656 nt	GQ425169<br>HV222<br>701 nt	GQ425213<br>HV222<br>857 nt	GQ425112<br>DML67438<br>1257 nt
Dictyota humifusa	JQ061096<br>SD712460<br>1023 nt	JQ061104<br>ODC1659<br>642 nt		JQ061117<br>ODC1659<br>701 nt	JQ061046<br>SD712460<br>859 nt	JQ061126<br>SD712066<br>1285 nt
Dictyota implexa	GQ425163<br>ODC1238<br>1173 nt	GQ425135<br>FS271<br>644 nt	GQ425140<br>LLGO249<br>656 nt	GQ425168<br>LLGO300<br>717 nt	GQ466076<br>ODC1238<br>874 nt	GQ425116<br>Kooistra1<br>1252 nt
Dictyota intermedia	GQ425165<br>TC1<br>825 nt	GQ425127<br>TC1<br>607 nt			EU395615<br>TC1<br>867 nt	DQ472086<br>TC1<br>1207 nt
Dictyota kunthii	GU290231<br>D104<br>1167 nt	GU290237<br>D102<br>598 nt	GU290245<br>D102<br>623 nt	GU290250<br>D102<br>717 nt	EU395618<br>D102<br>797 nt	DQ472057<br>D102<br>1246 nt

Taxon	LSU	*cox*1	*cox*3	*nad*1	*psb*A	*rbc*L
Dictyota liturata	GQ425159<br>KZN2282<br>1174 nt		GQ425144<br>HEC15721<br>537 nt	GQ425174<br>HEC15721<br>717 nt	GQ466075<br>HEC15816<br>877 nt	GQ425113<br>Sole1<br>1235 nt
Dictyota mediterranea	GU290233<br>SGAD1116<br>1172 nt	GU290236<br>SGAD1116<br>641 nt	GU290241<br>LLGO313<br>653 nt	GU290246<br>LLGO224<br>717 nt	GU255569<br>D653<br>861 nt	GU290254<br>D595<br>1247 nt
Dictyota mertensii	GQ425158<br>HV911<br>1172 nt	GQ425130<br>DR31<br>607 nt		GQ425180<br>DR31<br>709 nt	GQ425215<br>DR31<br>831 nt	GQ425114<br>DR32<br>1079 nt
Dictyota naevosa	DQ472108<br>KZNb2345<br>592 nt	JQ061105<br>KZNb2345<br>600 nt	JQ061109<br>KZNb2345<br>562 nt	JQ061118<br>KZNb2345<br>714 nt	EU395609<br>KZN2241<br>856 nt	DQ472084<br>KZN2241<br>1266 nt
Dictyota nigricans		JQ061106<br>D92<br>600 nt		JQ061119<br>D92<br>717 nt	JQ061068<br>D92<br>770 nt	DQ472077<br>D92<br>1250 nt
Dictyota pinnatifida	GQ425157<br>CLO31302<br>1166 nt	GQ425126<br>HV902<br>611 nt	GQ425142<br>HV932<br>619 nt	GQ425171<br>HV932<br>717 nt	EU395612<br>HV902<br>868 nt	GQ425115<br>Sole3<br>1239 nt
Dictyota rigida	GU290232<br>ODC1623<br>1104 nt	GQ425138<br>ODC1657<br>605 nt		GQ425181<br>ODC1657<br>709 nt	GQ466077<br>ODC1657<br>861 nt	GQ425117<br>ODC1623<br>1275 nt
Dictyota sandvicensis	DQ472118<br>ODC896<br>1051 nt	GU290239<br>ODC889<br>604 nt	GU290241<br>ODC889<br>404 nt	GU290248<br>ODC889<br>709 nt	JQ061078<br>ODC889<br>855 nt	DQ472063<br>ODC896<br>1242 nt
Dictyota spiralis	GQ425161<br>ODC1225<br>1173 nt	GU290235<br>ODC1071<br>635 nt			GQ466078<br>HEC15815<br>877 nt	DQ472074<br>ODC1029<br>1266 nt
Dictyota stolonifera	GQ425160<br>TZ0488<br>834 nt	GQ425139<br>TZ0488<br>598 nt		GQ425182<br>TZ0488<br>717 nt	GQ425222<br>HV819<br>870 nt	GQ425118<br>D264<br>1266 nt
Dilophus fastigiatus	DQ472123<br>D96<br>1166 nt	GQ425125<br>D96<br>518 nt		GQ425170<br>D96<br>717 nt	EU395614<br>D96<br>855 nt	DQ472068<br>D96<br>1263 nt
Padina arborescens	EU579996<br>435nt		AB358939<br>656nt		AY430357<br>883nt	AB358904<br>1237nt
Padina boergesenii	DQ472093<br>ODC890<br>1271nt					DQ472037<br>ODC890<br>1182nt
Padina crassa			AB358943<br>656nt		AY430361<br>701nt	
Padina japonica			AB358942<br>656nt		AY430360<br>701nt	AB358910<br>1137nt
Padina pavonica	EU580000<br>482nt		EU681454<br>656nt	EU681498<br>712nt	EU681649<br>844nt	EU579961<br>1193nt
Padina sanctae-crucis	DQ472092<br>CL030305<br>990nt		AB489969<br>656nt			DQ472036<br>CL030305<br>884nt

Taxon	LSU	*cox*1	*cox*3	*nad*1	*psb*A	*rbc*L
Rugulopteryx okamurae	GQ425149<br>D194<br>1099 nt	GQ425120<br>FS280<br>612 nt	GQ425147<br>FS280<br>657 nt	GQ425185<br>FS280<br>717 nt	AY748322<br>D194<br>867 nt	AB096888<br>D194<br>1017 nt
Scoresbyella profunda		GQ425121<br>DIC44<br>607 nt	GQ425148<br>DIC44<br>518 nt	GQ425186<br>DIC44<br>498 nt	EU395620<br>DIC44<br>855 nt	
Spatoglossum asperum	EU580003<br>494nt					EU579964<br>1193nt
Spatoglossum crassum					AY430355<br>883nt	AY422679<br>1292nt
